# Repeating Ponseti method for treating relapsed idiopathic congenital talipes equinovarus in children less than two years of age: a prospective study

**DOI:** 10.1186/s12891-025-08464-8

**Published:** 2025-03-15

**Authors:** Khalaf Fathy Elsayed Ahmed, Ashraf Rashad Marzouk, Marwan Shams Eldin

**Affiliations:** Orthopaedics and Traumatology Department, Sohag Faculty of Medicine, Sohag University, Sohag, Egypt

**Keywords:** Relapse, Idiopathic, Congenital talipes equinovarus, Repeating Ponseti method, Pirani score

## Abstract

**Background:**

Various conservative and non-conservative methods have been debated for their effectiveness in treating relapsed idiopathic congenital talipes equinovarus (CTEV). The aim of this work was the assessment of repeating Ponseti method in management of relapsed idiopathic CTEV in children less than two years of age.

**Methods:**

This prospective study was carried out on 50 clubfeet in 33 children aged 10 months - two years, who had relapsed idiopathic CTEV after documented complete correction of the initial deformity with Ponseti method and initiation of a bracing protocol, between April 2022 and April 2024. Before, during, and after the last cast removal, as well as at the end of follow-up, the Pirani scoring system was utilized to evaluate each clubfoot.

**Results:**

The mean age was 12.5 ± 6.08 months. Sex was male in 26 (52%) patients. The side was right in 28 (56%) patients. Pirani score was significantly lower after the last cast removal and at the end of follow up than before treatment (*P* < 0.001).

**Conclusion:**

Relapsed idiopathic CTEV in children less than two years of age were effectively managed with the repeating of Ponseti method.

**Type of study/level of evidence:**

Therapeutic IV.

**Trial registration:**

Not applicable.

**Cinical trial number:**

Not applicable.

## Background

Relapse of clubfoot or congenital talipes equinovarus (CTEV) refers to the reappearance of any aspect of the deformity requiring treatment after completing correction of initial deformities and initiation of a bracing protocol [1]. Non- compliance to bracing protocol, poorly-fitted braces, lower education and socioeconomic conditions of parents were reported as the most common reasons of relapsed CTEV [2–4].

Untreated or improperly treated relapses of clubfeet can lead to progressive transformation of dynamic and flexible deformities to more rigid ones with permanent disabilities. Early intervention is critical for successful outcomes [5].

Five relapse patterns of clubfeet have been recognized: Grade IA, decrease in ankle dorsiflexion from 15 degrees to neutral; Grade IB, dynamic forefoot adduction or supination; Grade IIA; rigid equinus; Grade IIB, rigid adduction of the forefoot/midfoot complex; and Grade III, combined two or more deformities, including fixed equinus, varus, and forefoot adduction [6].

However, several conservative and non-conservative therapies have been implemented to manage relapsed CTEV, the treatment approaches for relapsed CTEV still controversial [7–9].

To the best of our knowledge, we are unaware of any prospective study that investigated the assessment of revision of Ponseti method in management of relapsed idiopathic CTEV in children less than two years who were successfully treated previously with Ponseti method.

The aim of this work was the assessment of effectiveness of repeating Ponseti method in management of relapsed idiopathic CTEV following documented complete correction of the initial deformity with Ponseti method in children less than two years of age.

## Materials and methods

### Patients

This prospective study was carried out on 50 clubfeet in 33 children aged from 10 months − 2 years old, who had relapsed idiopathic CTEV after documented complete correction of the initial deformity with Ponseti method and initiation of a bracing protocol, between April 2022 and April 2024 in the orthoapedics surgery department of a tertiary university hospital. Different deformities were included as rigid forefoot adduction and cavus (grade IIB), and combined two or more deformities, including fixed equinus, varus, and forefoot adduction (grade III). Postural, syndromic, neurogenic talipes equinovarus, and post-surgical recurrences were excluded.

The study was done after approval from the ethical committee of Sohag faculty of medicine (IRB: Soh-Med-24-02-02PD). Informed written consents were obtained from the parents of the patients after explanation of the method of treatment. Participation was voluntary, and the subject was allowed to withdraw at any time. Data collection and analysis were done according to the declaration of Helsinki.

**Fig. 1:**
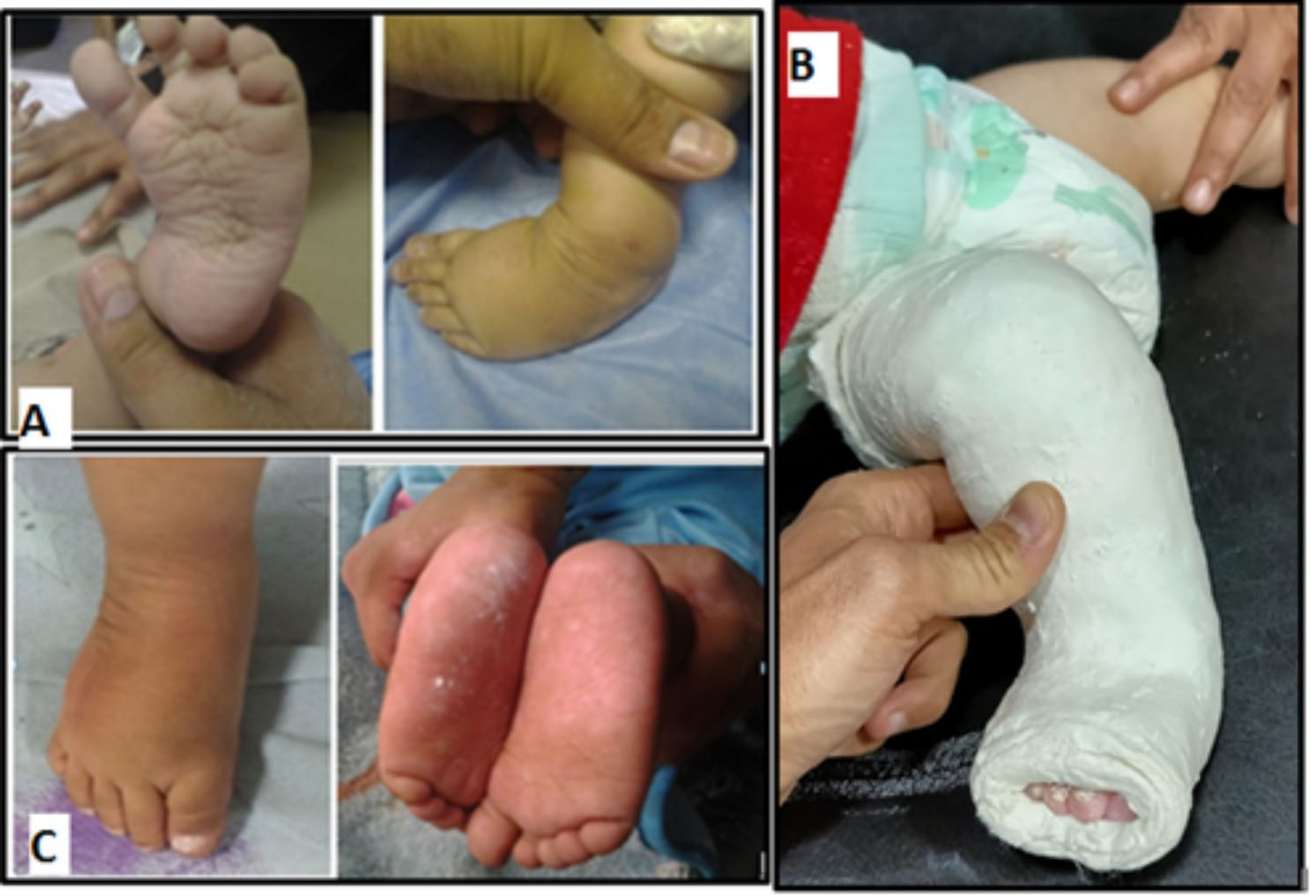
(**A**) Foot with relapsed clubfoot deformities before cast application, (**B**) Ponseti long leg cast application from toes to groin with knee flexed 90°, and (**C**) Desired correction after last cast removal

Detailed history was taken including searching for the reason of relapses trying to avoid re-relapses in the future. General examination was done to exclude atypical cases of CTEV. Assessment of each foot through physical examination was done to determine the extent and nature of the relapse. (Fig. 1A)

**Fig. 2:**
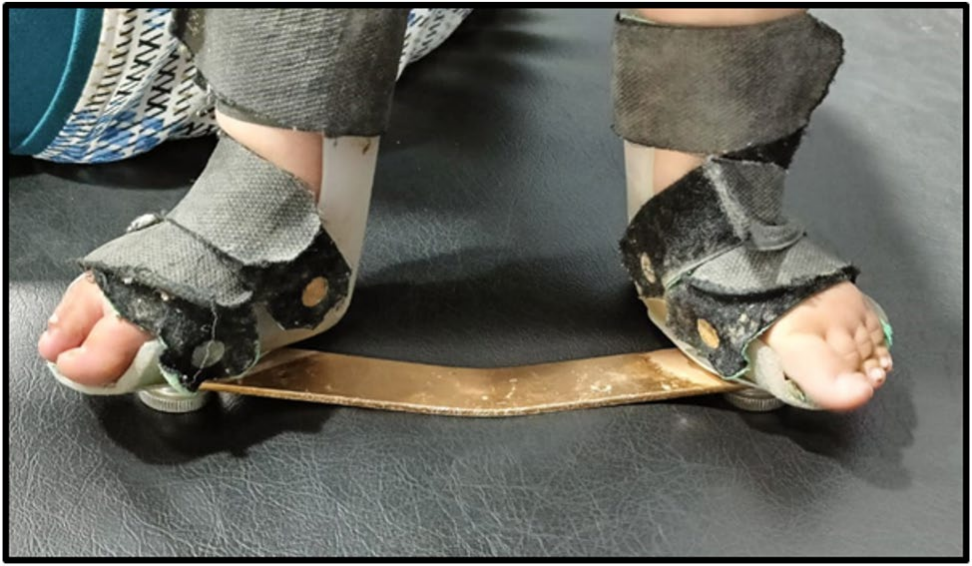
Foot abduction bracing with bar in between

We utilized the Pirani scoring system to evaluate the idiopathic clubfoot deformity, which assesses six clinical indicators of clubfoot, with three indicators evaluating midfoot contracture and three indicators evaluating hindfoot contracture. The hindfoot components included the posterior crease, empty heel, and rigid equinus. The midfoot components comprised the medial crease, curvature of the foot lateral border, and the talar head location. Each indicator was rated on a scale of 0 (no abnormality), 0.5 (moderate abnormality), or 1 (extreme abnormality). A cumulative score of 0 to 6 was assigned, with 6 indicating the most severe deformity [10].

Pirani scoring system is an essential clinical tool in evaluating the severity of CTEV deformities, monitoring the progress and effectiveness of treatment interventions, and guiding treatment strategies.

To achieve our objective, each foot was evaluated by the same orthopedic surgeon using the Pirani scoring system at the initial presentation. The same orthopedist scored each foot at each visit throughout treatment and follow-up, ensuring consistency and reliability.

### Methods

The repeating of the Ponseti method involved a series of manipulations and castings to correct the deformity, followed by using foot abduction brace to maintain the correction.

The Ponseti method was reapplied with gentle manipulation and serial above knee plaster castings with the knee flexed 90 degrees, with casts being changed every seven days. No possible risks upon the patient can be expected, except general risks from casting. Two to five casts were done to correct deformities, and then percutaneous achilles tenotomy under local anesthesia was performed to lengthen the tendon and allow for better correction in relapsed cases caused by tight achilles tendon and last casting was applied for one month (Fig. 1B).

After the desired complete correction was achieved, foot abduction bracing with 70 degrees external rotation was used to prevent further re-relapse. (Fig. 1C).

The standard Ponseti brace consists of shoes attached to a bar in 70 degrees external rotation, holding the feet in the corrected position. The brace was worn 23 h/ day for first consecutive three months then used at night (12 h/day) till age of four years. (Fig. 2).

Before, during, after the removal of the last cast, and at the end of follow up each foot was evaluated by Pirani scoring system.

All patients were followed up for at least one year.

### Statistical analysis

Statistical analysis was conducted using SPSS v26 (IBM Inc., Chicago, IL, USA). The unpaired Student’s t-test was applied to compare quantitative variables that were presented as means and standard deviations (SD). Qualitative variables, expressed in percentages and frequency, were analyzed using the Chi-square test. A two-tailed P value of less than 0.05 was considered to indicate statistical significance.

## Results

The study included 50 feet in 33 children (17 bilateral and 16 unilateral), the mean age was 12.5 ± 6.08 (range, 10–24) months. Sex was male in 26 (52%) patients and female in 24 (48%) patients. The side was right in 28 (56%) patients and left in 22 (44%) patients. The mean follow-up period was 12.63 ± 2.56 (range, 12–24) months. (Table 1)

**Table 1:** Demographic data of the studied children

Characteristics		Value
Total feet: children		50:33
Bilateral: unilateral		17:16
**Age (months)**		12.5 ± 6.08 (10–24)
**Sex**	**Male**	26 (52%)
	**Female**	24 (48%)
**Side**	**Right**	28 (56%)
	**Left**	22 (44%)
**Previous treatment method**	Ponseti method (100%)	
**Previous surgery**	None	
**Number of casts applied**	2–5	
**Follow up period (months)**	12.63 ± 2.56 (12–24)	

Data are presented as mean ± SD (Range) or frequency (%)

Pirani score was significantly lower after the last cast removal and at the end of follow up than before treatment (*P* < 0.001). (Table 2)

**Table 2:** Pirani score of the studied children

	Before treatment	After the last cast removal	At the end of follow up
Pirani score	5.5 ± 0.56	0.7 ± 0.24	1 ± 0.35
P-value		< 0.001*	< 0.001*

*: Significantly different as P value < 0.05. Data are presented as mean ± SD

## Discussion

The most important outcome in our study at the final follow up was Pirani score after the last cast removal and at the end of follow up was significantly lower than before treatment (*P* < 0.001). The lowering of Pirani score indicates effectiveness of repeating Ponseti method in improvement of relapsed deformities of idiopathic CTEV in children less than two years of age.

This finding was interpreted as Ponseti method gently corrects the deformity in stages, aligning the foot into a more normal position. As the foot’s alignment improves, the factors assessed in the Pirani score (like the midfoot score, and hindfoot score) show improvement [11]. The manipulations and castings increase the flexibility of the foot and ankle, reducing rigidity which is a key component assessed in the Pirani score [12]. Also, the Ponseti method effectively addresses the abnormal positioning of the heel and midfoot, which are crucial elements in the Pirani score [13]. The Ponseti method is non-invasive and gradual treatment, allowing for continuous assessment and adjustment. Each stage of treatment can lead to improvements in the score, reflecting the gradual correction of the deformity [8]. Additionally, it corrects the overall structure of the foot, not just the visible deformity. As the underlying structure improves, this is reflected in lowering of Pirani score [14].

Different scores have been used for evaluation of CTEV e.g. Pirani score, Dimeligo score, Goldner, and Walker classification [10, 15, 16]. Pirani score [10] was used in the current study because it is reliable and reproducible system and depends only on clinical data without any radiographic parameters as other scoring systems.

Liu et al. [17], compared repeated Ponseti method in treatment of relapsed clubfeet and cases without relapse by analysis of gait and kinematic gait deviation, and recommend repeated Ponseti method for treatment of relapsed clubfeet in the early stage.

Our results are comparable to the results of Rijal et al. [18], who found that Pirani score and midfoot contracture score were decreased after Ponseti treatment, Jain AK et al. [19], and Saini et al. [20] who found Pirani score improvement after Ponseti treatment of idiopathic CTEV.

In our study, there were 52% male and 48% female. Male predominance of CTEV has been reported in many of previous studies [7, 20–23].

In the current study, we evaluated clubfeet correction by clinical scores only without radiological investigations as there are several studies that had compared radiographic outcomes with clinical outcomes in clubfeet treated by ponseti method and showed that there was a statistically significant correlation of radiographic parameters and clinical outcomes, and thus radiographs can’t be used routinely in assessment and follow-up of these young aged children to avoid hazardous exposure to radiation [24–26].

The study was limited by several aspects including; a small sample size, its exclusive focus on a single center, and the comparatively short duration of patient follow-up. Further studies comparing our results with other techniques are required.

## Conclusion

Repeating of Ponseti technique is very effective in management of relapsed deformities of idiopathic CTEV in children less than two years of age.

## Data Availability

No datasets were generated or analysed during the current study.
